# Ultrasonographic detection of cranial cruciate ligament pathology in canine stifles without cranio-caudal instability

**DOI:** 10.18849/ve.v8i2.632

**Published:** 2023-05-05

**Authors:** Helen Tsoi+, Debra Canapp+, Sherman Canapp Jr.+

**Keywords:** MUSCULOSKELETAL ULTRASOUND, SPORTS MEDICINE, CRANIAL CRUCIATE LIGAMENT, ORTHOPAEDICS, ARTHROSCOPY, DOG, ARTHROTOMY, ARTHROSCOPY

## Abstract

**Abstract:**

**Objective:**

Explore the value of musculoskeletal ultrasound in detecting canine cruciate ligament pathology.

**Background:**

Partial tears of the cranial cruciate ligament (CCL) can be difficult to diagnose due to the lack of instability present on orthopaedic examination. Advanced diagnostics would be required for further evaluation. While a common tool in human medicine, magnetic resonance imaging (MRI) is of limited use in canines due to cost and the need for general anaesthesia. Musculoskeletal ultrasound (MSK-US) can be performed without anaesthesia but there are no current studies to date evaluating its usefulness in detecting partial tears of the cranial cruciate ligament.

**Evidentiary value:**

This is a retrospective case series of 32 dogs that underwent diagnostic MSK-US of the stifle who later had a surgical procedure (stifle arthroscopy / arthrotomy) to evaluate the intra-articular space.

**Methods:**

Medical records were evaluated between May 2014 – April 2020 for canines with clinically stable stifles that underwent both an MSK-US of the stifle followed by stifle surgery. Ultrasound findings of the CCL were compared to surgical findings.

**Results:**

Compared to surgical findings, ultrasound was a very sensitive test in detecting CCL pathology however it is less specific. Its sensitivity (SN), specificity (SP), positive predictive value (PPV) and negative predictive value (NPV) were 100%, 58.3%, 81.5%, and 100% respectively.

**Conclusion:**

MSK-US is a non-invasive test that can be performed with little to no sedation. Using a high frequency 18–5 MHz linear transducer, MSK-US is a sensitive test for detecting partial CCL injuries in dogs and may aid in its diagnosis for canines without stifle instability and be useful in guiding treatment. As it is less specific, arthroscopy or arthrotomy would be necessary to further confirm the intra-articular pathology.

**Application:**

Diagnostic MSK-US is a non-invasive tool that can be used to detect CCL pathology in the canine stifle. Its application can help guide treatment recommendations prior to a more invasive diagnostic / therapeutic procedure such as surgery or arthroscopy.

## Introduction

It has been well established that cranial cruciate ligament (CCL) disease is one of the most common orthopaedic conditions diagnosed in canines (Johnson et al., 1994; and Piermattei et al., 2006). Diagnosis is typically made by a positive cranial drawer (Jerram & Walker, 2003; Piermattei et al., 2006; De Rooster et al., 1998; Fossum, 2012; Johnson & Radasch 1988; and Widmer et al., 1994) or cranial tibial thrust test (De Rooster et al., 1998; Johnson & Johnson, 1993; and Slocum & Slocum, 1993) but may not be overtly evident in cases of incomplete tears which can make diagnosis of a partial CCL tear difficult (Piermattei et al., 2006; Fossum, 2012; Reed et al., 1995; and Strom, 1990). There are two bands of the CCL: the smaller craniomedial band, which is taut in both extension and flexion, and the larger caudolateral band which is taut in extension and lax in flexion (De Rooster et al., 1998; and Fossum, 2012). A tear of the craniomedial band may produce instability in flexion only and an isolated tear of the caudolateral band produces no instability (De Rooster et al., 1998; and Fossum, 2012). Increased periarticular and joint capsule fibrosis from chronic tears, patient size and temperament, location of tear (craniomedial versus caudolateral band), and other factors may contribute to the degree of instability detected on physical examination (Fossum, 2012; and Johnson & Johnson, 1993). In addition, puppies may have a normal stifle laxity present which can be confused as a ‘positive’ cranial drawer sign. This ‘puppy drawer’ typically has a distinct ‘end-feel’ when the ligament reaches the end of their stretch and usually only produces about 4–5 mm of movement (Jerram & Walker, 2003; Piermattei et al., 2006; Fossum, 2012; and Johnson & Johnson, 1993).

Distinction between what is normal and abnormal may not be intuitive and additional diagnostics are required to bring us closer to a diagnosis. Partial CCL tears have the ability to progress to a complete tear and a delay in treatment can lead to persistent lameness, progressive degenerative changes, the development of meniscus tears (Piermattei et al., 2006; Vasseur, 1984; Johnson & Radasch, 1988; Johnson & Johnson, 1993; Altarod et al., 2014; and Paatsama, 1988). Other diagnostic modalities are necessary to help aid in the diagnosis of cranial cruciate ligament tears particularly when instability is not apparent. Radiographs of the stifle may reveal displacement or obliteration of the infrapatellar fat pad secondary to increased joint effusion or thickening of the synovial lining, presence of osteophytes and other degenerative changes, and cranial displacement of the tibia in relation to the femur on stressed views in cases of a severe tear (Jerram & Walker 2003; Johnson et al., 1994; Vasseur, 1984; De Rooster et al., 1998; Johnson & Johnson, 1993; Rubin et al., 1975; and Marino & Joughin, 2010). Radiographs are also useful in ruling out other causes of lameness and instability such as avulsion fractures (Johnson & Johnson, 1993; and Marino & Loughin, 2010). They are however non-specific as the cruciate ligaments cannot be directly identified on radiography and stifle effusion and osteoarthritic changes may be present in other conditions affecting the stifle such as infectious disease, other non-infectious causes of arthritis, and neoplasia (Marino & Loughin, 2010). MRI has been shown to be accurate in the diagnosis of partial and complete cranial cruciate ligament disease in dogs (Marino & Loughin, 2010) but it is costly and requires the use of general anaesthesia, which limits their routine use in companion animals for the diagnosis of CCL pathology (Jerram & Walker, 2003; Fossum, 2012; and Johnson & Johnson, 1993). In contrast, MRI is a common diagnostic tool used to detect anterior cruciate ligament (ACL) injuries in humans (Johnson & Johnson, 1993; and Breukers et al., 2019). However, cost, availability, and requirement for the patient to lie still for an extended period of time are factors that still need to be considered (Breukers et al., 2019). Arthroscopy / arthrotomy has been discussed as the best practice for intra-articular evaluation with the potential for concurrent treatment but they are expensive and invasive (Din Dar et al., 2010; and Little et al., 2014).

The use of diagnostic musculoskeletal ultrasound (MSK-US) is uncommon in companion animals but it has been reported in the literature as a means of detecting intra-articular (IA) structures within the stifle (Reed et al., 1995; Kramer et al., 1999; Van der Vekens et al., 2019; Arnault et al., 2009; and Gnudi & Bertoni, 2001). In the normal stifle, a portion of the CCL can be seen as a hypoechoic band at the point of the tibial attachment (Reed et al., 1995; and Kramer et al., 1999) however, previous studies in canines have been unrewarding, concluding that tears in the CCL cannot be visualised (Little et al., 2014) or that detection rates are quite low (15.4–19.6% of cases) (Arnault et al., 2009; and Gnudi & Bertoni, 2001). These studies primarily evaluated complete tears of the CCL where there is likely a complete loss of fibre pattern in the region where they are typically found. In contrast, the use of arthrosonography in humans has been more rewarding in detecting anterior cruciate ligament pathology with sensitivities and specificities reported to be 88–91.9% and 80–100% respectively (Breukers et al., 2019; Grzelak et al., 2015; Henderson et al., 2015; Fuchs & Chylarecki, 2002; and Ptasznik et al., 1995). Skill and experience of an ultrasonographer as well as transducer frequency has been speculated to affect the accuracy and reliability of ultrasonographic findings (Van der Vekens et al., 2019; and Arnault et al., 2009).

In the hands of a seasoned ultrasonographer, MSK-US can be an invaluable tool for the diagnosis of soft tissue pathology as it is non-invasive and could potentially be performed under light to no sedation (Arnault et al., 2009; and Gnudi & Bertoni, 2001). To our knowledge, there is no published report in canines looking at the use of MSK-US in diagnosing partial tears of the cranial cruciate ligament. This retrospective study takes a look at the value of MSK-US in detecting cruciate ligament pathologies of canine stifles that display minimal to no instability when compared to arthroscopy or arthrotomy (Johnson & Johnson, 1993). We hypothesise that by looking at a subset of dogs in which we expect to have at least some intact fibres of the cranial cruciate ligament, that ultrasound would be able to detect pathology if it is present. By detecting partial tears early on, one may be able to intervene with alternative therapy such as regenerative medicine or surgical stabilisation sooner and thus protect the CCL from abnormal mechanical loading leading to further tearing and better preserve joint function (Fazio et al., 2018; Barker et al., 2016; and Canapp et al., 2016).

## Methods

### Case selection

Medical records of client-owned dogs were reviewed between May 2014 – April 2020 for cases that received diagnostic MSK-US of the stifle at a single institution (Veterinary Orthopedic & Sports Medicine, Annapolis Junction, Maryland). Data was collected for patients that had an MSK-US of the stifle which then later had a surgery (arthroscopy or arthrotomy) of the stifle to confirm the diagnosis. Information obtained included signalment, medical history, orthopaedic examination findings, diagnostics modalities performed (radiographs, MSK-US, etc.), and surgical findings (arthroscopy / arthrotomy). Cases were included if physical examination did not reveal the characteristic ‘cranial drawer / cranial tibial thrust’ or only mild instability was present. Cases were excluded from the study if there was moderate to severe instability, multi-ligamentous injuries (‘deranged stifles’), previous surgical intervention performed in the stifle, or other diagnoses made (medial patellar luxation, patellar tendon rupture). Some cases were evaluated for bilateral conditions or re-presented at a later date due to concerns for the contralateral limb. In these cases, each stifle was included individually.

### Orthopaedic examination

Data was collected for the limb affected, presence of stifle effusion, and presence of cranial drawer and tibial thrust in flexion and extension. Other orthopaedic or neurological exam findings were also recorded (iliopsoas discomfort, back / lumbosacral discomfort, patellar luxation). Documentation of additional diagnostics or treatments for the listed co-morbidities were not taken into account as the focus was placed on the ultrasonographic appearance of the cruciate ligaments for the purposes of this study.

### Diagnostic radiography

Lateral and cranio-caudal radiographs were evaluated for both stifles for the presence of joint effusion and stifle osteoarthritis as an indirect sign of intra-articular pathology. Additional images of pelvis and tarsi were present to rule out other causes of lameness.

### Diagnostic musculoskeletal ultrasound

**Figure 1:**
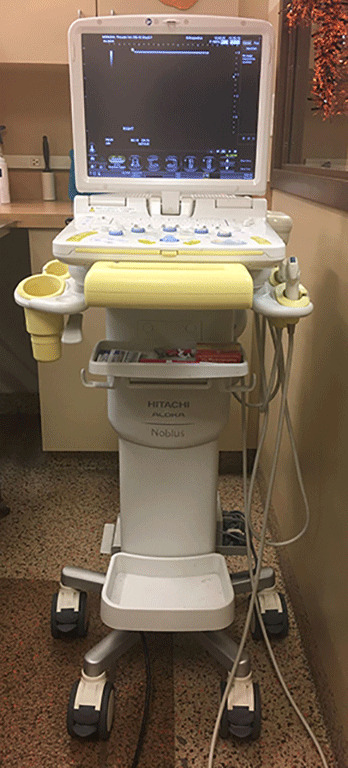
Ultrasound system (Noblus, Hitachi Aloka Medical, Twinsburg, OH).

MSK-US of the stifle was offered to patients in which a preliminary diagnosis of partial cranial cruciate ligament tear was made based on physical examination and radiographs. Ultrasonographic images were obtained by 38 mm, high frequency, linear transducer (18–5 MHz) (Figure 1).

**Figure 2:**
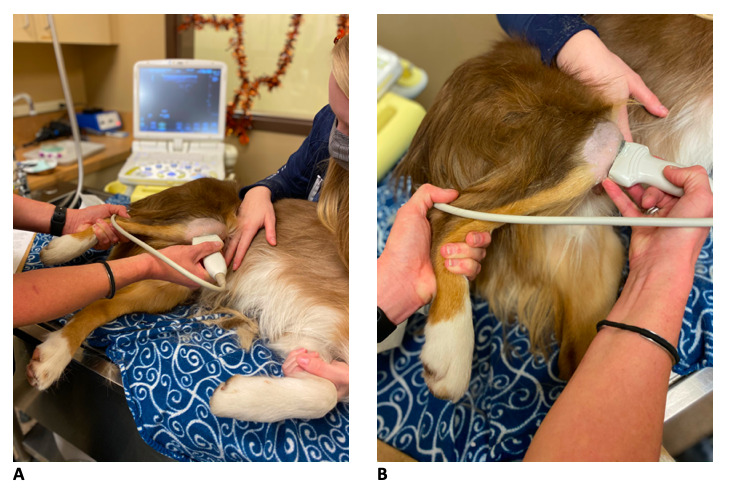
Positioning for optimal scan. (A) Patients are positioned in lateral recumbency with the stifle being scanned on top. (B) The stifle is placed in a fully flexed position to ‘open’ up the joint space.

Most patients did not require any sedation. Light sedation (dexmedetomidine (3 mcg/kg) + butorphanol (0.2 mg/kg) intravenous (IV) was used in those that were too anxious for an awake examination to be performed. Patients were placed in lateral recumbency with the stifle of interest on top (Figure 2A). The fur on the cranial, lateral, and medial aspects of the stifle were clipped from just proximal to the patella distally to the tibial crest. The skin was cleansed and saturated with 70% ethanol followed by the application of ultrasound coupling gel. The stifle is placed in a fully flexed position to ‘open’ up the joint space and improve visualisation of the intra-articular structures (Figure 2B).

With the stifle in deep flexion, the probe is placed longitudinally over the patellar region, with the patella proximal (towards left of monitor) and the tibia insertion distally (towards right of monitor). This position will reveal the patellar tendon and the intra-articular region to evaluate the fat pad, femoral bone margins, tibial crest / plateau bone margins, and cruciate ligament region. This view is used to assess for joint effusion, degenerative joint changes, and the presence of fibrotic / disruptive changes in the mid-body region of the cranial and caudal cruciate ligaments as well as the cranial cruciate ligament at its tibial insertion. To further evaluate the cruciate ligament region and obtain additional views of the cranial cruciate ligament (CCL) from tibial insertion, slightly turn the probe so that the proximal end moves laterally for an oblique approach to the femoral origin.

Echogenicity and fibre pattern were recorded for the cruciate ligaments and compared to the contralateral stifle. The changes were not categorised and defined into a formal grading scheme as the entirety of the ligaments cannot be fully viewed based on their position within the stifle. From the accessible views, findings were grouped into normal (which includes compensatory and age / work related changes) or abnormal (indicating cruciate ligament pathology).

The ultrasounds were performed by experienced ultrasonographers (two American College of Veterinary Sports Medicine and Rehabilitation (ACVSMR) Diplomates and two ACVSMR Residents).

### Surgical procedures of the stifle joint

Surgery reports were reviewed for cases that underwent a stifle arthroscopy or arthrotomy. A standard parapatellar approach to the stifle joint was utilised for portal placement during stifle arthroscopy or a routine mini-medial parapatellar arthrotomy was performed to the stifle joint for intra-articular evaluation. Data was recorded for the presence of pathology noted in the cruciate ligaments.

### Statistical analysis

Ultrasonographic and surgical findings of the cruciate ligaments were then compared and analysed to one another. The sensitivity (SN) and specificity (SP), positive predictive value (PPV) and negative predictive value (NPV) of MSK-US was determined by using surgical findings as the reference standard. The 95% confidence intervals were established using the ‘exact’ Clopper-Pearson method through an online calculator (MedCalc Software Ltd., 2023).

## Results

### Signalment

Thirty-two dogs met the inclusion criteria mentioned above. Table 1 displays the breeds represented in this study. The most common breed represented were Border Collie (8/32 (25%)), followed by Doberman Pinscher (3/32 (9.4%)), Labrador Retriever (3/32 [9.4%]), mixed breed (3/32 [9.4%]), Australian Shepherd (1/32 [6.3%]), Belgian Malinois (1/32 [6.3%]), Boxer (1/32 [6.3%]), Welsh Corgi (1/32 [6.3%]), Australian Cattle Dog (1/32 [3.1%]), Belgian Tervuren (1/32 [3.1%]), German Shepherd (1/32 [3.1%]), Great Dane (1/32 [3.1%]), Greyhound (1/32 [3.1%]), Leonberger (1/32 [3.1%]), and Samoyed (1/32 [3.1%]). The mean body weight was 27.1 kg (range: 12.5–59 kg) and the mean age was 4.3 years (range: 1.1–10.9 years) at the time of presentation. One patient’s date of birth was missing from the medical records and therefore was not included in the mean age calculation. Of these, 53.1% were male (five neutered, 12 intact) and 46.9% were females (10 spayed, five intact).

**Table 1:** Breeds represented in this study.

Breed	Frequency
Border Collie	8
Doberman Pinscher	3
Labrador Retriever	3
Mixed breed	3
Australian Shepherd	2
Belgian Malinois	2
Boxer	2
Welsh Corgi	2
Australian Cattle Dog	1
Belgian Tervuren	1
German Shepherd	1
Great Dane	1
Greyhound	1
Leonberger	1
Samoyed	1
Total	32

### Physical examination

The median duration of lameness was reported to be 2 months (range: 3 days – 2 years) at the time of presentation. All dogs were presented for a unilateral lameness. One of the dogs returned later for issues on its contralateral limb and therefore was counted twice as it met the inclusion criteria. The left hind limb was affected in 19 cases and the right hind limb was affected in 14 cases. Two dogs were presented for a unilateral lameness but had concurrent pathology in their contralateral stifles which were further explored during the same time period and therefore the limbs were also counted separately. When counting the evaluated stifles individually, 35 data points were available for analysis.

On an awake examination, 24/35 (68.6%) stifles had no clinical signs of cranio-caudal instability as evidenced by a positive cranial drawer or cranial tibial thrust test. Of those, two had an increase in internal rotation of the stifle and one had a positive caudal drawer test. Eleven stifles exhibited mild instability in flexion only. Three of those had concerns for medial instability and one had an abnormal movement of the fibular head. All (35/35 [100%]) of the affected limbs had palpable stifle effusion.

The most common concurrent pathology reported was iliopsoas (18/35 [51.4%]) discomfort or tightness on the affected limb. Additional examination findings included lower lumber / lumbosacral discomfort (8/35 [22.8%]) and sensitivities over the piriformis region (3/35 [8.6%]) of the affected limb.

### Pelvic limb radiographs

Stifle radiographs were obtained for all cases. In the affected limb, the most common findings were increased stifle effusion (33/33 [100%]) and degenerative / osteoarthritic changes (8/33 [24.2%]). Joint effusion was apparent in (14/33 [42.4%]) of the contralateral stifles and degenerative changes present in (3/33 [9%]). One dog had evidence of mild bilateral hip dysplasia and two dogs had some remodeling seen at the region of the greater trochanter. Four dogs had remodeling of the lumbosacral junction on lateral spine radiographs.

### Stifle ultrasound and surgical findings

**Figure 3:**
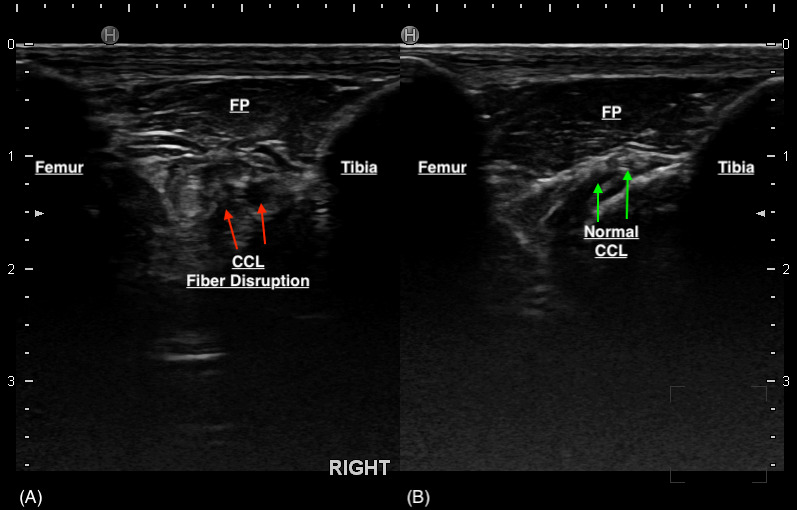
(A) CCL deficient stifle. The red arrows represent the regions of disruption to the CCL fibres. There is displacement of the patellar fat pad (FP). (B) Normal stifle. The green arrows point to an intact CCL.

The majority (80%) of the ultrasounds were performed by one individual (DC), the remainder were performed by ACVSMR residents. The median time between ultrasound findings and surgery was 1 day (range: 0 –125 days). Only a portion of the CCL can be visualised with sonography due to its position within the stifle. This portion is its distal insertion on the tibial tuberosity and is suspected to be approximately 30–60% of the CCL as reported in a previous study (Van der Vekens et al., 2019). Ultrasonographic evaluation determined that there was CCL pathology in 28/35 cases. Of these cases, CCL pathology was defined by hyperechoic fibre changes in 96.4% of the cases, fibre disruption in 39.3%, and calcification around the tibial insertion of the CCL in 14.3% of cases (Table 2). Six stifles were determined to have an intact CCL on ultrasound and the CCL was unable to be visualised in one. The majority of cases had hyperechoic changes, but it was not uncommon to have a mixture of lesions (hyperechoic fibre changes ± fibre disruption ± calcification around the tibial insertion) (Figure 3). Other stifle lesions documented with ultrasonography were not analysed as this paper was intended to focus on the appearance of cranial cruciate ligament disease.

**Table 2:** Description of lesions noted on diagnostic MSK-US.

Lesion Description	Number of cases
Hyperechoic change	27
Fibre disruption	11
Irregular bony margins/calcification around the tibial insertion of CCL	4

**Figure 4:**
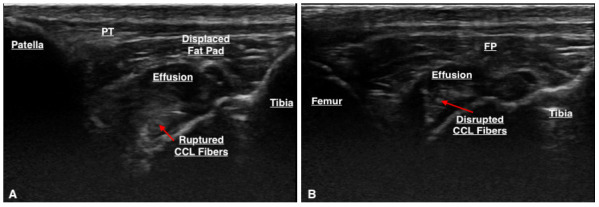
(A & B) The arrows point to regions of CCL fibre disruption and the presence of hyperechoic changes surrounding a hypoechoic structure.

**Figure 5:**
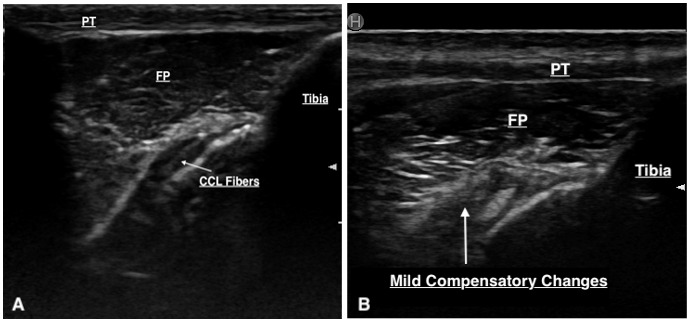
(A) Hypoechoic structure representing a normal, intact CCL. (B) Mild hyperechoic changes without evidence of fibre disruption.

Thirty-three stifles underwent arthroscopy for joint exploration and two underwent arthrotomy. Disruption to the CCL was identified in 22 stifles (partial tear [n = 21] and complete tear [n = 1]). Ultrasound detected changes to the CCL (echogenicity, fibre disruption and / or mineralisation) in all 22 stifles (Figure 4). Stifles that were considered normal or had ‘compensatory / age-related’ changes showed a hypoechoic structure determined to be the intact CCL or mild hyperechoic changes without overt fibre disruption, respectively (Figure 5). Ultrasound reported a partial tear of the CCL in 5/35 (14.3%) stifles which were not identified intra-operatively, however three of these stifles were reported to have caudal cruciate ligament (CdCL) pathology at the time of surgery. In one stifle, the CCL could not be visualised due to the presence of an echogenic, mass-like structure speculated on ultrasonography which was not confirmed via arthroscopy. This stifle was not included in statistical testing.

**Figure 6:**
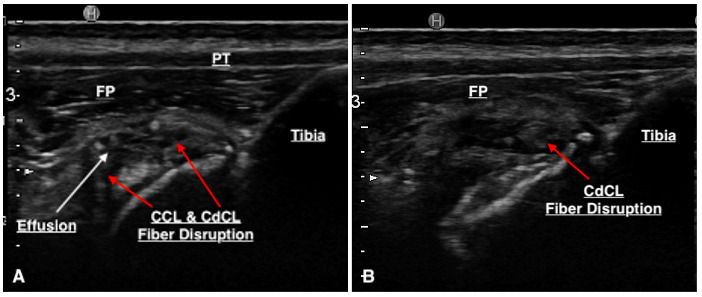
(A) A region of a hypoechoic structure surrounded by hyperechoic changes seen cranial to the CCL suspected to represent the CdCL. (B) Another ultrasound image identifying CdCL pathology.

Sensitivity (SN), specificity (SP), positive predictive value (PPV) and negative predictive value (NPV) of ultrasonography for detecting CCL pathology were 100% (95% CI 84.6%, 100%), 58.3% (95% CI 27.7%, 84.8%), 81.5% (95% CI 69.3%, 89.6%), and 100% (95% CI 100%) respectively. This was calculated using the table below (Table 3). In 15/35 stifles, disruption to the CdCL (three complete, 12 partial) was diagnosed on arthroscopy. Ultrasound suspected changes to the CdCL in 11 of these cases (Figure 6). Ultrasound reports did not consistently comment on the CdCL as techniques and experiences of our ultrasonographers have been reported to evolve over time.

**Table 3:** Cranial cruciate ligament pathology detected by ultrasound and surgery.

		Surgery	
		Pathology detected	Not detected
Ultrasound	Pathology detected	22 (True positive)	5 (False positive)
	Not detected	0 (False negative)	7 (True negative)

## Discussion

Historically, previous studies have reported that ultrasonographic examinations for the canine stifle have not been rewarding for the diagnosis of cranial cruciate ligament tears in dogs (Kramer et al., 1999; Arnault et al., 2009; and Gnudi & Bertoni, 2001). While earlier reports of sonographic examinations of normal stifles report that the cranial cruciate ligament (CCL) can be routinely observed via ultrasound, it can be speculated that once there is a complete tear, visualisation of the CCL is impaired as the ligament is no longer present in its typical location (Reed et al., 1995; Arnault et al., 2009; Gnudi & Bertoni, 2001; and Cauvin & Smith, 2019). In addition, only 30–60% of the total ligament fibre at the distal insertion can be visualised on ultrasonography given its position within the stifle (Van der Vekens et al., 2019). Our study focused on cases in which we did not suspect to have a complete rupture of the cruciate ligament and used a higher frequency transducer (18 MHz) than what was previously reported in the literature (7.5–16 MHz) (Reed et al., 1995; Kramer et al., 1999; Arnault et al., 2009; and Gnudi & Bertoni, 2001). Ultrasound detection of CCL pathology was very high with a sensitivity reported at 100% in this study. This difference compared to previous studies may be due to the fact that the presence of intact fibres in a partial CCL tear makes it easier to observe given its more consistent location within the stifle compared to a complete tear. A previous report describing CCL tears confirm their diagnosis by observing a truncated hypoechoic line near the surface of the cranio-proximal tibial (Gnudi & Bertoni, 2001). It is possible that they were having a difficult time isolating this hypoechoic structure as the normal hypoechoic structure of the CCL begins to appear more hyperechoic when there is disease present. In our experience, the lack of observable CCL fibres in its usual location coupled with echogenicity changes typically represent a severe or complete rupture of the CCL. However, in the majority of cases, cranio-caudal stifle instability is present, therefore the use of ultrasound to diagnose the rupture is not warranted. The higher frequency probe may also allow us to see finer detail that otherwise may not be detected with a lower frequency transducer. Another explanation for this is that our study does have a high selection bias as we selected for patients that had pathology that was suspected to be localised to the knee without obvious instability. If no pathology was detected during ultrasound, arthroscopy was not typically recommended. Owners were also less likely to agree to a more invasive procedure (i.e., surgery) if the ultrasound was inconclusive. Due to this, we did not have many ‘normals’ that could provide as a control which would have strengthened our data. In our study, MSK-US for detecting CCL pathology has a lower specificity (54.5%) as evidenced by the larger number of false positives obtained. Changes present in nearby structures such as the synovium or the CdCL may be mis-interpreted to represent changes to the CCL. Although surgical assessment of the CdCL were provided in some cases, we did not attempt to interpret the results of the testing as inconsistencies on reporting would make it difficult to draw any valid conclusions. One of the ultrasonographers (DC) report that it was only recently within the past 2 years that they began to notice changes in a certain area, distinct from the CCL insertion on the tibia (Figure 6) that appeared to correlate with CdCL changes seen on arthroscopy. Earlier examinations were less likely to have scanned this region or provide an interpretation specific to CdCL pathology. Previous studies have acknowledged the possibility of visualising the CdCL with ultrasonography (Reed et al., 1995; and Kramer et al., 1999) while others denote that it cannot be visualised (Arnault et al., 2009). Reed et al. (1995) described the ultrasonographic location of the CdCL to be seen at the lateral surface of the medial condyle on its point of attachment on the femur while Kramer et al. (1999) report that it can only be visualised in larger dogs on a transverse section. In our study, we do not scan the stifles using these methods described above. Anectodally, the changes observed (Figure 6) are present cranial to the CCL which is inconsistent with its anatomic location within the stifle. However, this may support an indirect sign of CdCL pathology, but further studies would be required to explore this significance. This further denotes that experience and technique of an ultrasonographer plays a large role in the diagnostic value of MSK-US.

Our study design compared ultrasound findings to surgical findings (arthroscopy / arthrotomy) which we considered the reference standard. While surgery provides direct visualisation of the joint, limited views of the stifle anatomy can be seen intra-articularly (Fazio et al, 2018; and Cauvin & Smith, 2019). It has been suggested that since the cruciate ligaments are extra-synovial, arthroscopy provides a limited surface view of the ligament and may underestimate the pathology present even with careful probing (Fazio et al., 2018). Histology would be required to confirm intra-substance lesions that are not present grossly on visual examination through arthroscopy or arthrotomy (Van der Vekens, 2019; and Doring et al., 2018). Unfortunately, histologic evaluation is not possible with this type of clinical study. If ultrasound was detecting some of these earlier changes that is not being visualised during surgery, it could also lead to the number of false positives obtained during this study when comparing ultrasound to surgical findings. However, it is unclear whether these findings are of any clinical significance at this time to warrant surgical stabilisation or treatment, however they may prove useful as a means of monitoring progression of disease in a clinically stable stifle.

Several limitations are present in this study. This study was retrospective and therefore subjected to variations in technique, reporting, and incomplete documentation. The lack of standardised reporting for arthroscopic / surgical findings for intra-articular structures does have an effect on the results. Mild fibre changes, degeneration or disruption to the ligaments may still be translated to the report as intact if torn fibres were not visualised or they were unable to see the entirety of the cruciate ligaments within the joint. As discussed previously, there is a selection bias present due to the nature of cases selected. We also did not have any controls for comparison. Surgery would not be recommended if pathology was not detected, contributing to a verification bias which is demonstrated by our high sensitivity and low specificity. This is further confirmed by the 100% confidence interval obtained for the sensitivity and negative predictive value which does not occur.

The ultrasounds were performed by four different people which may add to the variability of the lesions detected due to the differences in experience level, although the majority of the scans were performed by one individual (DC). The range of years that we looked at were also quite broad (2014–2020) and the range of technique and confidence of an individual can vary tremendously throughout that time period and affect the findings reported. Timing between ultrasound and surgery may also influence the results reported. The majority (24/33 [72.7%]) of patients had surgery performed within 72 hours of their ultrasound date, however some patients were not scheduled for surgery until weeks or months after their ultrasound procedure. The significance of the time gap is unclear at this time although one may expect a progression of the disease process that may be inaccurately represented by earlier diagnostics. To our knowledge, there are no studies looking into this comparison. A previously published study in human medicine reported a high sensitivity and specificity in US imaging for anterior cruciate ligament (ACL) tears despite a longer time gap between imaging and surgery (median: 5 weeks; range 0–48 weeks) (Breukers et al., 2019). The significance of time on the effect and appearance of the ACL was not explored or discussed in this study.

What would have strengthened our study is a prospective design in which patients undergoing stifle surgery were enrolled to receive a stifle ultrasound irrespective of suspected stifle pathology. This would decrease or eliminate selection or verification bias in the samples. Blinding of the veterinary surgeon to physical exam findings and the results of diagnostic testing would not likely be possible as in clinical practice, veterinary surgeons rely on the use of supporting diagnostics to make recommendations for surgery. The ultrasonographer may be blinded to the physical examination findings and the results of other diagnostic testing. A prospective study would also be able to standardise and minimise the timing between ultrasonography and surgery, reducing the influences of a longer time gap.

In humans, ultrasound is capable of detecting partial and complete tears of the ACL and they are even incorporating the combination of direct, indirect, and dynamic tests of ACL rupture during ultrasonography to strengthen its diagnostic value (Breukers et al., 2019; Ptasznik et al., 1995; Fuchs & Chylarecki, 2002; Wang et al., 2018; Kumar et al., 2018; and Palm et al., 2009). The development of additional techniques may also prove valuable in canine CCL detection. In one study, Seong et al. (2005) suggested that the use of intra-articular (IA) saline during ultrasonography can improve visualisation of the CCL however these patients underwent general anaesthesia for the procedure. Unfortunately, literature on arthrosonography in small animals is lacking.

Other considerations are to study interobserver variability to look at the influence of experience on the ultrasound findings as it has been reported to be highly operator dependent (Coss, 2019; and Han et al., 2008). Based on the angle and orientation in which one holds the probe, a different image and echogenicity can be created from the same structure (Reed et al., 1995) which can contribute to misleading interpretations of the images.

This study explored the capability of ultrasound to detect cranial cruciate ligament pathology in dogs. This paper focused on stifle injuries without overt instability as the presence of craniocaudal laxity is pathognomonic for CCL rupture making further diagnostics redundant for diagnosis. Ultrasound is a non-invasive tool and the images can be interpreted at the same time of the scan. It can also be performed on an awake patient or with minimal sedation. This is in contrast to an MRI which requires general anaesthesia. Our study showed that MSK-US is a highly sensitive test in detecting partial CCL ruptures in canines. CCL pathology detected on ultrasound warrants further exploration. On the other hand, if CCL pathology is not detected, a more invasive procedure such as surgery may potentially be avoided.

## Author contributions

**Helen Tsoi**: Conceptualisation, Methodology, Validation, Formal analysis, Investigation, Resources, Data curation, Writing – Original draft, Writing – Review & Editing, Visualisation, Project administration. **Debra Canapp**: Conceptualisation, Methodology, Validation, Investigation, Resources, Data curation, Writing – Review & Editing. **Sherman Canapp**: Conceptualisation, Validation, Resources, Writing – Review & Editing.

## ORCiD

Helen Tsoi: https://orcid.org/0000-0001-8158-739X Sherman O. Canapp Jr.: https://orcid.org/0000-0002-0271-3413

## Conflict of Interest

The authors declare no conflicts of interest.
